# The transformative journey of chronic myeloid leukemia

**DOI:** 10.2217/ijh-2018-0008

**Published:** 2018-08-28

**Authors:** Pratap Neelakantan, Adam Mead

**Affiliations:** 1 Royal Berkshire Hospital, Reading, UK; 2 NIHR Biomedical Research Centre, University of Oxford, Oxford, UK; 3 Medical research Council Molecular Haematology Unit, WIMM, University of Oxford, Oxford, UK

Five thousand years after the ancient physicians began conceiving treatment methodologies for cancers in the form of arsenic, the publication of the results of the pivotal IRIS trial with imatinib for chronic myeloid leukemia (CML) in 2003 was a remarkable landmark [1]. Imatinib, one of the first examples of a so called ‘magic-bullet’ therapy, transformed the outlook of a cancer which, up to that point, had a median survival of 3–5 years from diagnosis [2]. Indeed, since CML was the first described in 1845 by JH Bennett in Edinburgh, the causative genetic lesion of which the Philadelphia (ph) chromosome was discovered 140 years later by J Rowley, this disease has been at the forefront of advances in diagnosis and treatment of cancer. Most patients between the years 1970–2000 needed bone marrow transplantation to offer any realistic hope of long-term survival and this was a procedure restricted to a minority of patients and also associated with significant toxicity. The discovery of the Philadelphia chromosome fast tracked the pace of discoveries in the field, to the extent that the first targeted therapy for this disease was being tested in first-in-man trials during the late 1990s, culminating in the remarkable results of the IRIS trial [1]. For the first time, patients were achieving extremely high rates of sustained cytogenetic remissions [1].

Fast forward 10 years and newer ‘2nd generation’ tyrosine kinase inhibitors (TKI) were further transforming the outlook for patients with CML. These newer 2nd generation TKIs were able to induce faster and deeper responses and also offered the minority of patients who fail imatinib, an alternative treatment [3]. Indeed, such is the success of the current therapeutic armamentarium that recent studies have shown that the life expectancy for CML patients is now approaching that of the normal population, a remarkable turnaround over a relatively short period of time [2]. The goals of therapy in CML have been completely rewritten and are now focused on the achievement of faster and deeper molecular responses which correlate with better outcomes for patients.

Although TKIs are highly-effective treatments, until recently the conventional wisdom was that therapy needed to be ‘lifelong’, an important consideration as TKIs are not without side effects. The next chapter in the CML story started with publication of the French STIM study, which suggested that it was possible to successfully stop TKI therapy without disease recurrence in approximately half the patients who achieve deep molecular responses [4]. Subsequently, large prospective clinical trials of the 2nd generation TKI nilotinib have confirmed these findings, leading to this drug receiving the first licence for ‘treatment-free remission’ [5,6]. Today, It is hard to believe that one of the key goals of therapy is now to achieve deep molecular remission so that treatment-free remission and ‘cure’ might be a reality for many patients in routine clinical practice. Although hurdles remain, in particular to increase the proportion of patients who achieve such deep molecular remissions, if the CML story continues along its previous trajectory, we are well on the way toward achieving the dream of cure for the majority of patients with CML.
